# Complete genome sequence of a new *Saccharothrix* sp., NBAS 001, isolated from soil sample

**DOI:** 10.1128/mra.00572-26

**Published:** 2026-06-15

**Authors:** Jia-Zhen Sun, Ling Chen, Wei Chen, Xiaoyan Liu, Lei Zhu

**Affiliations:** 1 National Biopesticide Engineering Research Centre, Hubei Biopesticide Engineering Research Centre, Hubei Academy of Agricultural Sciences 117996 , Wuhan, China; DOE Joint Genome Institute, Berkeley, California, USA

**Keywords:** *Saccharothrix*, complete genome sequence, novel species, soil, actinomycetes, secondary metabolites

## Abstract

This study reports the complete genome sequence of *Saccharothrix* sp. NBAS 001, a strain isolated from soil near the shore of Nanhu Lake in Wuhan, Hubei Province, China. The genome comprises a single circular chromosome of 10,561,717 bp and encodes 9,388 CDS.

## ANNOUNCEMENT

The genus *Saccharothrix*, a group of filamentous actinobacteria widely distributed across diverse natural habitats, is well recognized for its capacity to synthesize a variety of bioactive secondary metabolites, yet genomic resources for this group remain limited (1, 2). Strain NBAS 001 was isolated in December 2023 from soil (5–10 cm depth) sampled near the shore of Nanhu Lake, Wuhan, Hubei Province, China (30.48309° N, 114.35181° E). Soil samples (10 g) were serially diluted and spread on mannitol soya flour (MS) medium plates supplemented with nalidixic acid (20 μg/mL) and cycloheximide (100 μg/mL) (3). A single colony was obtained after 7 days of incubation (30°C) and purified by streak-plating on MS plates. To resolve its taxonomic status and expand available genomic data, this strain was subjected to whole-genome sequencing.

The purified colony was inoculated into MS medium and cultured at 30°C to the mid-logarithmic growth phase. Genomic DNA was extracted using the E.Z.N.A. Bacterial DNA Kit (Omega Bio-tek, USA) (yielding 6.5 μg). For library preparation and sequencing, 1.0 μg (short reads) and 3.0 μg (long reads) of DNA were used. DNA concentration was assessed using a NanoDrop 2000 UV-Vis spectrophotometer (Thermo Fisher Scientific, USA). For short-read sequencing, genomic DNA was fragmented to an average size of 300–500 bp using a Covaris M220 focused ultra-sonicator. Libraries were prepared using the NEBNext Ultra DNA Library Prep Kit (NEB, USA), with target fragments recovered via 2% agarose gel electrophoresis. After quantification with a TBS-380 fluorometer (PicoGreen; Invitrogen, USA), sequencing was performed on the DNBSEQ-T7 platform (MGI, China) using a 150 bp paired-end strategy. Raw data were processed using Trimmomatic v0.36 (ILLUMINACLIP:adapters.fa:2:30:10 SLIDINGWINDOW:4:15 MINLEN:75) to remove low-quality reads, yielding 7,451,333 short reads with a total length of 1,117,700,000 bp (Q30 = 95.69%). For long-read sequencing, libraries were constructed using the CycloneSEQ library preparation protocol (4) and sequenced on the CycloneSEQ-WT02 platform (MGI). Raw data were filtered via NanoFilt v2.8.0 (-q 7, -l 50), and statistical analysis of sequencing data was performed using SeqKit v0.12.0. This process yielded 172,746 long reads totaling 1,528,058,635 bp, achieving a read N50 of 15,258 bp and an average length of 8,845 bp. Hybrid assembly was executed via Unicycler v0.5.0 (5). Assembly integrity was confirmed by evaluating GC-depth distribution and sequencing coverage consistency.

The assembly yielded a complete circular chromosome of 10,561,717 bp with a G + C content of 72.35% (Table 1). The circularity was confirmed by the identification of overlapping sequences at the assembly termini. The genome was annotated via NCBI PGAP v6.10, identifying 9,388 CDSs, 65 tRNAs, 12 rRNAs, and 3 ncRNAs (6). The 16S rRNA gene sequence showed 99.2% similarity to *Saccharothrix espanaensis* DSM 44229^T^ (HE804045). Further taxonomic analysis via JSpeciesWS v5.0.3 and TYGS revealed that strain NBAS 001 shared an average nucleotide identity of 88.5% and a digital DNA-DNA hybridization value of 40.9% with this type strain. These values are well below the established species demarcation thresholds (7, 8), suggesting that strain NBAS 001 represents a novel species of the genus *Saccharothrix*. All software was run with default parameters unless otherwise specified.

**TABLE 1:** Genome features of strain NBAS 001

Feature	Data for chromosome
Assembly length (bp)	10,561,717
G + C content (%)	72.35
No. of assembled contigs	1
No. of coding sequences	9,388
No. of tRNAs	65
No. of rRNAs (5S, 16S, 23S)	12 (4, 4, 4)
Protein-coding genes annotated with NR	9,006
Protein-coding genes annotated with GO	1,146
Protein-coding genes annotated with eggNOG	7,142
Protein-coding genes connected to KEGG pathways	3,212
Protein-coding genes annotated with Swiss-Prot	4,276
Protein-coding genes annotated with CAZy	288
Protein-coding genes annotated with CARD	187

## Data Availability

This Whole Genome Shotgun project has been deposited in GenBank under accession no. JBWVLX000000000. The version described in this paper is the first version, JBWVLX000000000.1. The raw sequencing reads have been deposited in the Sequence Read Archive under accession numbers SRR38292091 (DNBSEQ) and SRR38292090 (CycloneSEQ). The BioProject and BioSample accession numbers are PRJNA1430991 and SAMN56295768, respectively.
